# Joint Stiffness Is Heritable and Associated with Fibrotic Conditions and Joint Replacement

**DOI:** 10.1371/journal.pone.0133629

**Published:** 2015-07-21

**Authors:** Frances M. Williams, Nicholas S. Kalson, Stella M. Fabiane, Derek A. Mann, David J. Deehan

**Affiliations:** 1 Department of Twin Research & Genetic Epidemiology, King’s College London, London United Kingdom; 2 Department of Orthopaedics, The Royal Victoria Infirmary and the Freeman Hospital, Newcastle upon Tyne, United Kingdom; 3 Fibrosis Research Group, Institute of Cellular Medicine, University of Newcastle, Newcasltle upon Tyne, United Kingdom; University of Sheffield, UNITED KINGDOM

## Abstract

**Objective:**

Joint stiffness is a common, debilitating, age-related symptom, which may be seen after total joint replacement (TJR). Stiffness also occurs in fibrotic conditions such as shoulder capsulitis and Dupuytren's contracture. We speculated that the two traits (TJR and fibrotic disease) are linked pathogenically.

**Methods:**

Using the TwinsUK NIHR BRC BioResource we tested the hypotheses that 1) joint (hip and knee) stiffness, TJR (hip and knee), and fibrotic conditions are associated and 2) genetic factors contribute to them.

**Results:**

Participating twins (n = 9718) had completed self-reported questionnaires on the traits of interest. All three traits were significantly associated with increasing age and body mass index (BMI), as well as female sex, on univariate analysis. Multivariable logistic regression analyses showed a significant association between TJR and joint stiffness (OR = 3.96, 95% confidence interval, CI 2.77–5.68) and between fibrotic conditions and joint stiffness (OR = 2.39, 1.74–3.29), adjusting for age, sex, BMI and twin relatedness. Monozygotic versus dizygotic intraclass correlations gave heritability estimates for TJR = 46% and joint stiffness = 32%.

**Conclusion:**

That fibrotic conditions, joint stiffness and TJR are significantly associated suggests a common disease process, possibly fibrosis, which is genetically mediated.

## Introduction

Joint stiffness is a common, debilitating symptom that significantly affects quality of life. Total joint replacement (TJR) is the treatment of choice for end stage, painful, stiff joints, usually the result of osteoarthritis. More than 90,000 total knee replacements are performed each year in the UK [1]. A number of fibrotic diseases are known to affect joints and connective tissue giving rise to the symptom of joint stiffness, including frozen shoulder [2], arthrofibrosis [3] and Dupuytren’s contracture [4]. Joint stiffness may also develop spontaneously [2] or following an insult such as trauma or surgery [5]. The contribution of genetic factors to the symptom of joint stiffness has not, to our knowledge, been explored previously, although a contribution of genetic factors to frozen shoulder has been shown [6]. The process of fibrosis involves the deposition of a dense, disorganised extracellular matrix of collagen [7]. It is likely that different triggers converge on a common ‘fibrotic pathway’ involving α-smooth muscle actin containing myofibroblasts, TGF-1βsignaling and rapid deposition and tensioning of the new matrix [8].

TwinsUK is the UK's largest registry of monozygotic (MZ) and dizygotic (DZ) twins. It contains extensive genotype and phenotype data obtained at clinical visits and by mailed and online questionnaires. The twin characteristics have been shown to be similar to the general singleton population for a range of traits and lifestyle factors [9]. TwinsUK has contributed to the genetic understanding of a wide variety of traits and diseases including musculoskeletal disease. We examined existing information in TwinsUK from self-reported questionnaires to test the hypotheses that 1) joint stiffness, TJR, and fibrotic conditions (shoulder capsulitis and Dupuytren's contracture) are associated with one another; and 2) genetic factors contribute to the traits.

## Methods

Participants were selected from the TwinsUK registry [10] on the availability of data from four different questionnaires and clinical visits between 1992 and 2008. Responses to the questions ‘Have you ever had pain or stiffness in the following joints? left knee/right knee/left hip/right hip’; ‘have you undergone a total knee or total hip replacement?’; and ‘been diagnosed with frozen shoulder or Dupuytren’s contracture?’ (fibrotic condition) were extracted. Age, sex and body mass index (BMI) were also extracted for the time-point relevant to each questionnaire. Non-respondents for each particular condition were considered not to have the condition, and were coded as negatives. This standard data-handling practice in the TwinsUK dataset precludes descriptive analysis of ‘non-responders’. Participants were not aware of a specific hypothesis related to joint stiffness or joint replacement being tested in this study, nor was the temporal relationship of the traits explored. Ethics committee approval for the study was obtained from St Thomas’ Hospital Ethical Review Board. All participants gave written, informed consent. King’s College Hospital approved the consent procedure.

### Statistical analysis

Logistic regression analysis was used to determine the association between the three traits of interest, joint stiffness, TJR, and fibrotic conditions, adjusting for age, sex, BMI and the twin relationship. Heritability estimates were calculated by comparing intraclass correlations in MZ versus DZ twins for TJR and joint stiffness (frozen shoulder was reported previously). For the purposes of this analysis, missing data were assumed to be negative, thus biasing the study towards the null. Statistical analysis was performed using Stata software (StataCorp, Texas, USA).

## Results

The sample comprised 9718 twins having information on at least one trait at any time-point (knee or hip joint stiffness, fibrotic condition, TJR; Table 1). This included 174 twins reporting a TKR and 258 twins having a THR. Frozen shoulder was reported in 747 cases (7.7%) and Dupuytren’s contracture in 28 (0.3%), with a prevalence of fibrotic conditions overall of 7.8%. The mean age of the sample was 47.2 years (range 19–75, SD 13.8 years, MZ 47.1, DZ 47.3) and 87.8% were female. Two hundred and eighty seven (2.9%) respondents reported joint stiffness at either the knee or hip. The variable TJR (Total Joint Replacement) is a summary of TKR and/or THR; some individuals reported both conditions. The same applied to reported joint stiffness (hip and/or knee stiffness) and fibrotic conditions (frozen shoulder and/or Dupuytren’s contracture). There was no significant difference in age or BMI between the MZ and DZ twins. The complete dataset used in this study can be found in the file S1 Appendix.

**Table 1:** Characteristics of the TwinsUK sample, by zygosity.

	Monozygotic (%of total)	Dizygotic (% of total)	Total
**Total respondents**	5200	4518	9718
Mean age (years)	47.1	47.3	47.2
Mean BMI (kg/m^2^)	25.3	25.3	25.3
No of females (%)	4517 (46.5)	4012 (41.3)	8529 (87.8)
Stiff knee, N (%)	92 (0.9%)	131 (1.3%)	223 (2.3%)
Stiff hip, N (%)	57 (0.6%)	85 (0.9%)	142 (1.5%)
Stiff joint (total), N (total, either hip or knee or both) (%)	121 (1.2%)	166 (1.7%)	287 (2.9%)
Frozen shoulder, N (%)	333 (3.4%)	414 (4.3%)	747 (7.7%)
Dupuytren's contracture, N (%)	15 (0.2%)	13 (0.1%)	28 (0.3%)
Fibrotic condition, N (total, either Dupuytren’s or frozen shoulder or both) (%)	340 (3.5%)	422 (4.3%)	762 (7.8%)
TKR N (%)	93 (1.0%)	81 (0.8%)	174 (1.8%)
THR N (%)	126 (1.3%)	132 (1.4%)	258 (5.3%)
TJR N (total, either hip or knee or both) (%)	188 (1.9%)	190 (2.0)	378 (3.9%)

Demographic characteristics of the TwinsUK sample. There were no significant differences in age, BMI or sex between the zygosity groups. TKR represents total knee replacement; THR, hip replacement; TJR, total joint (either knee or hip) replacement. N is number.

### Univariable analysis

Increasing age and BMI were found to be significant risk factors for both joint stiffness and TJR. Twins reporting a fibrotic condition were more likely to complain of hip or knee joint stiffness (60/762 (7.9%) versus 227/8956 (2.5%), p = <0.001, Pearson χ^2^ test). Of those twins reporting joint stiffness and/or a fibrotic condition (989 out of 9718, 10%), 95 had undergone a TJR, compared with 283 out of 8729 without fibrosis or joint stiffness (9.6% vs 3.2%, p = <0.001, Pearson χ^2^ test). Univariable logistic regressions were also used to model the magnitude and significance of the associations (Table 2) and showed similar findings, even with adjustment for sex and twin relatedness. Joint stiffness was positively significantly associated with TJR (OR 5.32, 95%CI 3.78–7.49).

**Table 2:** Results of uni- and multivariable logistic regression analyses.

	Joint stiffness		Fibrotic condition		TJR	
	Univariable	Multivariable	Univariable	Multivariable	Univariable	Multivariable
**Age**	1.03 (1.03–1.04)	1.03 (1.02–1.04)	1.05 (1.04–1.05)	1.04 (1.04–1.05)	1.07 (1.06–1.08)	1.07 (1.06–1.08)
**BMI**	1.05 (1.02–1.07)	1.04 (1.01–1.06)	1.03 (1.01–1.05)	1.01 (1.00–1.03)	1.06 (1.04–1.08)	1.04 (1.02–1.06)
**Joint stiffness**	-	-	3.12 (2.29–4.26)	2.39 (1.74–3.29)	5.32 (3.78–7.49)	3.96 (2.77–5.68)
**Fibrotic condition**			-	-	2.01 (1.49–2.69)	1.32 (0.95–1.83)

Odds ratios and 95% confidence intervals between traits of interest, outcome variable shown in top row, all analyses adjusted for sex and twin relatedness. The univariable analyses included the predictor variable of interest in the left hand (LH) column relating to a given cell; the multivariable analyses included all the predictors LH column with values filled in. Thus for TJR, joint stiffness is highly associated (OR—3.96) in a model that includes joint stiffness, fibrotic condition, BMI, age, sex and twin relatedness.

### Multivariable analysis

Results of regression analyses considering all traits together are also shown in Table 2. Respondents with TJR were significantly more likely to report joint stiffness (3.96, 95% CI 2.77–5.68) after adjusting for age, sex, BMI, fibrotic condition and twin relatedness. The association between TJR and fibrosis remained positive but was not quite statistically significant (OR 1.32, 95% CI 0.95–1.83). Respondents were more likely to have a fibrotic condition (frozen shoulder or Dupuytren’s) if they reported knee or hip stiffness (OR 2.39, 95% CI 1.74–3.29, Table 2). Removing Dupuytren’s disease and analysing those with frozen shoulder alone provided a similar strong association with joint stiffness (OR 2.39, 95% CI 1.75–3.28, data not shown).

### Heritability of joint replacement and joint stiffness

Comparison of the intraclass correlations in MZ versus DZ twins for TJR and joint stiffness is shown in Table 3. The low prevalence of TJR in our data did not allow formal modelling of heritabilities, nor a bivariate analysis of the traits to determine shared genetic factors. TJR and joint stiffness showed higher intraclass correlation in MZ vs DZ twins, with heritability estimates of 46% and 32% respectively (Table 3). Fibrotic conditions had a heritability of 28%.

**Table 3:** Case-wise concordance, intraclass correlations and heritabilities for joint stiffness, fibrotic conditions and TJR.

		N concordant pairs	N discordant pairs	Case-wise concordance^a^	Intraclass correlation (95% CI)	Estimated heritability
Joint stiffness	MZ	25	71	0.41	0.40 (0.28–0.51)	**0.32**
	DZ	22	122	0.26	0.24 (0.14–0.33)	
Fibrosis	MZ	52	224	0.32	0.27 (0.20–0.34)	**0.28**
	DZ	43	327	0.21	0.13 (0.07–0.18)	
TJR	MZ	32	118	0.35	0.33 (0.24–0.42)	**0.46**
	DZ	12	159	0.13	0.09 (0.02–0.16)	

^a^Case-wise concordance calculated as *2Nc/(2Nc+Nd)*, where *Nc* is the number of concordant pairs and *Nd* the number of discordant pairs. The crude heritability was estimated from intraclass correlations: *2(corr*_*MZ*_ *-corr*_*DZ*_ *)*. N represents number. The 95% CIs were calculated using the bootstrap method.

## Discussion

The symptom of joint stiffness is linked with various pathologies and is not predictable. We have explored the relationship between stiffness, fibrotic conditions and the propensity to large joint replacement, based on the notion that genetic influence may modulate the susceptibility to fibrosis. We have shown, for the first time, an association between fibrotic conditions (frozen shoulder, Dupuytren's contracture), joint stiffness and TJR, which is evident in this large dataset, even after adjusting for known risk factors, such as age and BMI [11]. Furthermore, the three to four fold increase in intraclass correlation in MZ compared to DZ twins for TJR and joint stiffness is suggestive of a genetic influence on a common underlying disease process affecting connective tissues. Unfortunately, the sample size did not allow bivariate modelling of the genetic and environmental predisposing factors.

A number of different factors contribute to the decision to replace a joint, including total loss of joint space on plain radiograph, as a marker of endstage osteoarthritis. Knee osteoarthritis itself also has a significant genetic component, with heritability estimates of ~40% [12]. A sibling study of severe osteoarthritis requiring TJR had a heritability estimate of 27–31% [13]. A similar heritability estimate has been described previously for frozen shoulder using the TwinsUK registry [6] and the point prevalence of Dupuytren’s disease found in the present study is similar to that previously reported in the UK [14].

This was an initial, observational study and there are several limitations. Connective tissue research is limited by accurate disease definition. Diagnosis of arthrofibrosis and frozen shoulder remains difficult and the self-reported data included pain, as well as stiffness. However, the incidence of fibrotic conditions here is similar to that reported previously [2, 6] and the twins, on the whole, live separately and complete the questionnaires without reference to the co-twin [10]. Any missing data were assumed to be negative (non-respondents for each particular condition were considered not to have the condition), making it likely that incidence values are, if anything, underestimates. This data handling approach biases our dataset to the null making positive findings less, rather than more, likely. There is no evidence that such bias would differentially affect MZ rather than DZ twins. Another limitation of the study is that age, BMI and questionnaire data came from different time points, although each was appropriate to the questionnaire used. We were unable to adjust for conditions (surgery or trauma) that predispose to joint stiffness, nor did we consider joint replacement secondary to fracture. However, the significant associations and heritabilities make the conclusions drawn justified within the limitations of the data.

Treatment options for fibrotic joint conditions are severely limited at present, and include stretching or surgically removing the fibrotic tissue; they do not address the biological basis of disease. This limited physical approach to the tissue may contribute to a recurrence of fibrosis, which is frequently seen [3]. A better understanding of the biomolecular basis of joint stiffness and the processes underpinning fibrosis would allow the development of targeted pharmaceutical treatment. That joint stiffness and TJR are heritable suggests a significant genetic component to the disease process, and ideally a bivariate analysis would allow consideration of shared genetic influence. This degree of heritability is comparable to diseases, such as breast cancer [15]. The findings in TwinsUK need replication in an independent sample but could lead to screening for fibrotic conditions prior to TJR. Further work to identify the genetic variants contributing to fibrosis would facilitate the use of personalised medicine in TJR surgery.

## Supporting Information

S1 Appendix Raw data set used in the study. The entire dataset used in this study is presented in the file S1 Appendix.
(XLSX)
